# Leveraging large language models for heuristic usability assessment of medical software: Insights with the Radiation Planning Assistant

**DOI:** 10.1002/acm2.70495

**Published:** 2026-02-18

**Authors:** Laurence E. Court, Jacobus Smit, Lourens Strauss, William Shaw, Andrea Marais, Christoph Trauernicht, Nanette Joubert, Elaine Smith, Shona Badre, Graeme L. Lazarus, Thekiso Khotle, Lauren Netherton, Wanda van Heerden, Carlos Cardenas, Monica Serban, Jan Seuntjens, Christine V. Chung, Pavel Govyadinov, Meena Khan, Saurabh Nair, Tucker Netherton, Lifei Zhang

**Affiliations:** ^1^ Department of Radiation Physics The University of Texas MD Anderson Cancer Center Houston Texas USA; ^2^ Department of Medical Physics University of the Free State School of Medicine Bloemfontein South Africa; ^3^ Division of Medical Physics, Tygerberg Hospital Stellenbosch University Cape Town South Africa; ^4^ Department of Radiation Medicine, Groote Schuur Hospital University of Cape Town Cape Town South Africa; ^5^ Department of Radiation Oncology Inkosi Albert Luthuli Central Hospital Durban South Africa; ^6^ Department of Radiation Oncology, Charlotte Maxeke Johannesburg Academic Hospital Wits University Johannesburg South Africa; ^7^ Houston Texas USA; ^8^ Icon Oncology Johannesburg South Africa; ^9^ Department of Radiation Oncology University of Alabama at Birmingham Birmingham Alabama USA; ^10^ Department of Radiation Oncology Princess Margaret Cancer Centre Toronto Canada

**Keywords:** Heuristic evaluation, Large language models, Usability, User interface design

## Abstract

**Background:**

Usability engineering is essential for ensuring the safety and effectiveness of medical software, as design‐related issues are a leading cause of use errors in clinical settings. Heuristic evaluation provides a practical approach to identifying usability problems, but its outcomes depend heavily on expert interpretation. Large Language Models (LLMs), such as ChatGPT, offer a potential means to augment heuristic evaluation by generating structured, context‐aware usability feedback. This study explored the use of ChatGPT to support heuristic assessment of the Radiation Planning Assistant (RPA), a web‐based radiotherapy planning tool designed to support clinical teams in low‐ and middle‐income countries.

**Methods:**

ChatGPT was provided with the RPA user and technical guides, training videos for each functional dashboard, and Zhang et al.’s 14 usability heuristics. The model was instructed to score each dashboard according to these heuristics, using Zhang's 0–4 severity scale, and to propose concrete interface improvements. The resulting feedback was reviewed and scored independently by the RPA developer team and by 13 users during a dedicated User Meeting. Comparative analysis was performed between ChatGPT, developer, and user ratings.

**Results:**

ChatGPT identified 26 potential usability issues across six heuristic domains. The developer team considered nine of these actionable, though all were classified as minor (severity ≤ 2). User ratings showed wide variability, with nine suggestions achieving mean scores ≥ 1.5. Qualitative agreement between users and developers was limited, underscoring the importance of diverse perspectives in heuristic evaluation. Three suggestions—enhanced upload logs, reversible actions (“reopen request”), and stronger error prevention—were rated as potentially high priority by a minority of users. ChatGPT's ratings were consistent across dashboards.

**Conclusions:**

While ChatGPT did not reveal any critical usability failures, its heuristic assessment proved valuable in prompting discussion, identifying minor refinements, and enriching both developer and user engagement with the RPA's interface design. This study demonstrates that LLMs can serve as an effective, low‐cost complement to conventional heuristic evaluation, supporting early‐stage usability review and stakeholder dialogue in the development of medical software.

## 1 INTRODUCTION

Usability engineering systematically applies human factors principles to the design and evaluation of medical device interfaces.^1^ It is a vital component of medical device development because deficiencies in interface design have been consistently identified as leading contributors to use errors, surpassing hardware and software failures in their impact on patient safety. Such errors often arise from ambiguous displays, poorly structured workflows, or non‐intuitive controls that misalign with users’ mental models and clinical contexts. Consequently, rigorous usability evaluation throughout the design and validation process is essential to ensure that medical devices support accurate task performance, mitigate the risk of human error, and enhance overall system reliability in clinical practice.^1^, ^2^, ^3^, ^4^ An understanding of usability engineering is vital not only for commercial developers but also for those creating in‐house software, which is increasingly common in radiation oncology.^5^, ^6^, ^7^

One usability engineering approach is that of heuristic evaluation. Heuristic evaluation is a cost‐effective usability assessment method that facilitates the timely identification of major usability issues in a product.^8^ The heuristic evaluation process typically involves three or more evaluators systematically reviewing the interface, applying a set of usability heuristics, documenting violations, and rating the severity of each issue. Its application to the evaluation of medical device interfaces, including software, is well documented and is among the usability testing approaches recommended in IEC 62366‐1. The primary reference for this method is the work of Zhang et al.,^8^ who, building on earlier research, developed a set of 14 heuristics designed to detect usability problems that may contribute to medical errors. A summary of these heuristics is provided in Table 1. Heuristics have previously been applied to many devices, including radiotherapy devices.^9^, ^10^, ^11^, ^12^, ^13^

**TABLE 1:** Heuristics for evaluation of medical interfaces, adapted from Zhang et al.^8^

Heuristic	Description
Consistency and standards	Interfaces should adhere to established conventions and standards so that similar actions and terms carry consistent meanings.
Visibility of system state	The system must continuously provide users with clear feedback about its current state or progress.
Match between system and the real world	System design should align with real‐world concepts and user expectations, using familiar formats and language.
Minimalist design	Only essential information should be presented to minimize distraction and avoid overwhelming the user.
Minimize memory load	Users should not be required to memorize information across different screens; critical data should be readily accessible.
Informative feedback	Every action should result in timely and meaningful feedback to help users understand system responses.
Flexibility and efficiency	Interfaces should support both novice and expert users, offering shortcuts or customization where appropriate.
Good error messages	Error messages should be clear, informative, and guide users toward resolution without creating confusion.
Error prevention	Systems should be designed to minimize the chance of user errors occurring in the first place.
Clear closure	Each task should have a clear beginning and end, with unambiguous indications of completion.
Reversible actions	Users should be able to reverse or undo actions, reducing the risk associated with mistakes.
Use users’ language	Terminology and information should be presented in the language familiar to the intended users, avoiding unnecessary jargon.
Users in control	Users should remain in control of system interactions and not feel forced into unintended actions.
Help and documentation	Support resources, such as instructions or documentation, should be easily accessible when needed.

Although straightforward to perform, heuristic evaluation may benefit from additional feedback from a large‐language model (LLM). Large Language Models (LLMs) are advanced AI systems trained on vast amounts of text data that can produce structured responses, integrate knowledge, and generate contextually appropriate insights. They have shown potential across many applications,^14^, ^15^ although they can have some important limitations such as model hallucinations, bias, and lack of explainability.^16^, ^17^ In this work, we applied a large‐language model (LLM) to heuristic analysis of a radiotherapy treatment planning support software, the Radiation Planning Assistant.^18^ This tool has undergone extensive risk assessment using FMEA and other techniques,^19^, ^20^, ^21^ showing that user error (data entry) is often the most likely failure mode—thus emphasizing the importance of usability engineering to reduce risk. Here, ChatGPT is used to score usability, following Zhang, and to provide concrete suggestions for improvements to the user interface. These suggestions were then separately scored and reviewed by the developer team and a group of RPA users.

## 2 METHODS

### 2.1 The Radiation Planning Assistant

The Radiation Planning Assistant (RPA 1.0.0.5) is a web‐based tool for automated contouring and radiotherapy planning, designed specifically to support clinical teams in low‐ and middle‐income countries, helping them scale their efforts to treat more patients with high‐quality radiotherapy plans.^22^, ^23^, ^24^ It combines a variety of different technologies, including deep learning (autocontouring) and knowledge‐based planning (volume‐modulated arc therapy [VMAT] optimization). The RPA was developed using a software‐as‐a‐service framework to minimize operational costs and enhance system robustness. This web‐based architecture eliminates the need for local installation or maintenance, facilitates seamless software updates with minimal customization, and ensures reliable performance across diverse clinical environments. The RPA was designed, tested, and iterated following usability principles, including using the Zhang heuristics, as well as risk assessment (FMEA) and hazard testing,^19^, ^20^ with many user interface improvements already made prior to clinical release. The RPA has now been in clinical use for over 1 year.

**FIGURE 1:**
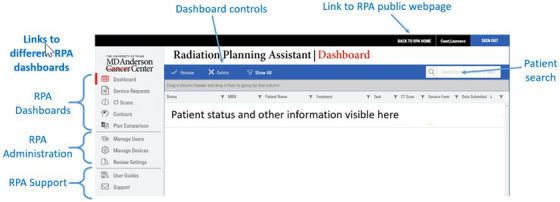
Screenshot of the main dashboard of the RPA, as seen in Google Chrome (Google LLC) on a standard monitor.

The complete workflow for generating a radiotherapy plan using the RPA is described in Court et al.^18^ Users interact with the RPA only through its dashboards (Figure 1), each of which supports a specific task (such as uploading a CT scan). To start a case, the user submits a service request containing the physician's prescription and selected treatment technique, and then uploads the patient's CT dataset. The RPA then automatically generates contours and creates a preliminary treatment plan.)

The completed plan is exported in DICOM format and downloaded from the RPA platform. It is then imported into the institution's treatment planning system, where the dose is recalculated to match the local delivery machine. The treatment planner and radiation oncologist may make further adjustments as needed. The plan then follows the institution's standard clinical workflow, including peer review and full quality assurance before treatment delivery.

### 2.2 ChatGPT interactions

The following files were uploaded to *ChatGPT* (GPT‐5.0 model; OpenAI, San Francisco, CA, USA): (1) RPA User Guide, (2) RPA Technical Guide, (3) Training videos for each process/dashboard (one video for each process listed below), and (4) Zhang heuristics paper.^8^

The following instructions were then given to ChatGPT:

1. Review the uploaded user guide and technical guide for context.
2. Review the newly uploaded video of the dashboard/module:
3. Extract 10 evenly spaced frames throughout the video.
4. Save them as images.
5. Include them in an Appendix of the report.
6. Score the dashboard/module against Zhang et al.’s 14 usability heuristics using the usability scoring metric from Zhang et al (Table 2)
7. For each heuristic scored 3 or 4, include:
8. A detailed explanation of the usability problem.
9. Concrete improvement suggestions (what to add/change/remove).

**TABLE 2:** Usability scoring, from Zhang et al.^8^

Score	Description
0	Not a usability problem at all
1	Cosmetic problem only. Need not be fixed unless extra time is available
2	Minor usability problem. Fixing this should be given low priority
3	Major usability problem. Important to fix. Should be given high priority
4	Usability catastrophe. Imperative to fix before product release

This process was then repeated using the exact same prompts to assess the usability for the following dashboards and processes in the RPA, using the same instructions each time:

- Main dashboard
- Service request
- CT upload
- Contour upload
- Plan comparison
- User and device management

### 2.3 Feedback and scoring by the developer team and RPA users

Suggestions for improvement provided by ChatGPT were then examined in detail by the developer team (LEC, PG, SN, CC, MK). This review verified that ChatGPT had accurately interpreted the screenshots and manuals and that the proposed improvements were appropriate. Duplicates were consolidated/removed. The developer team scored the feedback and provided comments on the ChatGPT feedback.

Finally, a meeting of RPA users was held at the annual congress of the South African Association of Physicists in Medicine and Biology (Durban, October 2025). At that meeting, current and potential RPA users, clinical physicists, and others reviewed the ChatGPT feedback. Each feedback item was discussed as a group, followed by independent scoring by each user on a scoresheet (see Appendix). Developer and user scores and feedback were compared. Specifically, consensus developer scores and average user scores (and percentage of users who scored 3 or higher).

## 3 RESULTS

### 3.1 ChatGPT scoring

A summary of the usability scores, according to ChatGPT are shown in Table 3. In total, ChatGPT provided 26 suggestions for improvement. These are detailed in the following tables. Of these, 25 were applicable to the main dashboard, 11 to the Service Request dashboard, 16 to the CT scan upload dashboard, 6 to contour upload, 4 to plan comparison, and 1 to user device/management.

**TABLE 3:** Summary of the usability scores, according to ChatGPT. Items identified as high priority (score of 3 or above) are highlighted in red.

Heuristic	Main dashboard	Service request	CT scan	Contour upload	Plan comparison	User and device management
Consistency	2	2	2	2	2	2
Visibility	3	3	3	3	3	3
Match	2	2	2	2	2	2
Minimalist	2	2	2	2	2	2
Memory	2	2	2	2	2	2
Feedback	3	3	3	3	3	3
Flexibility	2	2	2	2	2	2
Message	2	2	2	2	2	2
Error	2	2	3	2	2	2
Closure	3	3	2	3	2	3
Undo	3	3	3	3	3	3
Language	2	2	2	2	2	2
Control	3	3	3	3	3	3
Document	1	1	1	1	1	1

### 3.2 Developer and user team feedback

The Development Team consensus scoring and comments are provided in the tables below. They did not score any of the suggestions as 3 or 4.

**FIGURE 2:**
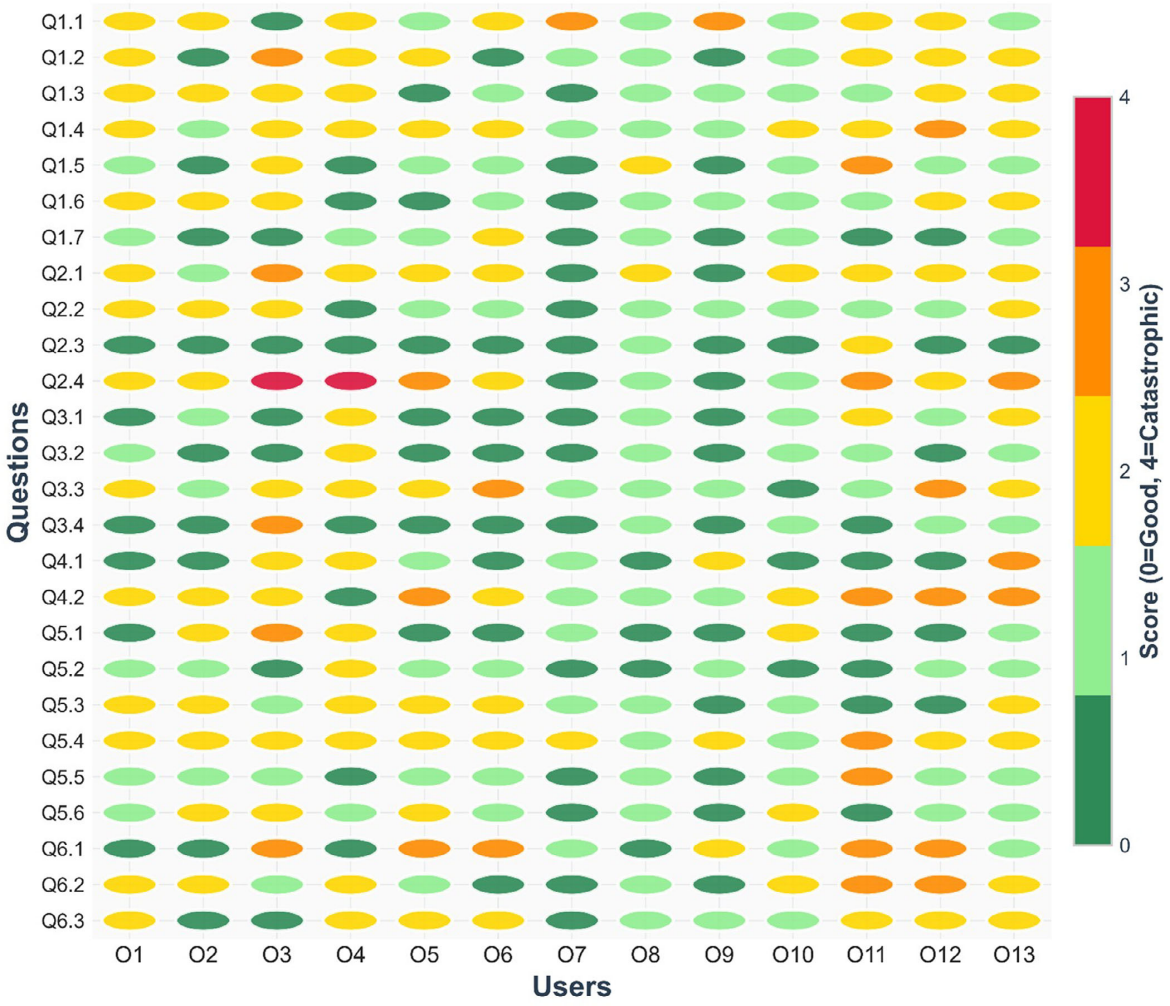
Rater scores for each ChatGPT suggestion. The response of each user to each question can be seen, showing there was significant inter‐rater variability throughout this study.

Thirteen raters completed score sheets at the User Meeting, and nine of them reported having experience with the RPA. Two raters had less than 5 years of clinical experience, two had between 5 and 10 years, and nine had more than 10 years of experience. The group included one oncologist, one engineer, and eleven medical physicists, four of whom were heads of department. Qualitatively, the range of rater scores was large, as can be seen in Figure 2. Raters with no experience and with experience using the RPA gave comparable scores (average 1.3 ± 0.6 vs. 1.1 ± 0.5).

There were no ChatGPT suggestions for which most raters scored a 3 or more. Three or more raters (23%) scored 3 or more (i.e., change should be prioritized) for three suggestions (12%), across three usability heuristics:

- Informative feedback: Offer download/view of upload logs for transparency (Table 5). Users expressed the need for logs in case of audit. Logs are kept but have to be shared by the service team.
- Reversible actions: Add a ‘reopen request’ action that restores prior state and logs the change (Table 7). The users shared a need for a simpler approach for repeat planning (e.g., with a different fractionation). Currently this task would involve several tasks on different dashboards (i.e., copy CT, then approve, and copy Service Request and approve).
- Prevent errors: Block progression with clear, actionable preflight checks (Table 7). Users indicated a wish to prevent some actions that are currently allowed—for example if the user places a reference point in an inappropriate point on the CT, they would like the RPA to warn the user rather than simply allowing them to continue.

The developer team (prior to the User Meeting) separately scored each of these suggestions as 0, indicating their opinion that they are not usability issues.

The heuristics for which ChatGPT scored 3 or 4 are reviewed in detail below, including a review of the definition of the heuristic, according to Zhang et al.,^8^ review suggestions provided by ChatGPT, and responses from the developer team (consensus score) and users (average of individual scores and percentage of users who scored a 3 or 4) – Tables 4, 5, 6, 7, 8, 9. Comments are generally the consensus comments that came from group discussions between the developers or users.

**TABLE 4:** Visibility of system state: Suggestions and responses.

	ChatGPT suggestions	Developer comments	Developer group score	User comments	Average user group score	% scores ≥3
1.1	Add a persistent global status area (system health, queue length)	It may be useful to show the system is operating normally, or if certain services are down (e.g., preventing autocontouring). Queue length might be useful if queues get long, or if the user needs a quick turnaround (perhaps for an adaptive plan)	2	If you have many users, it may be very helpful. If the system is down, something should prevent me from using it. Perhaps a red banner.	1.7	15%
1.2	Display real‐time indicators (spinners, timestamps, percentage complete)	We have a percentage complete bar for CT upload, but no other functions. We could add, if users find they are frustrated by the lack of this information.	2	If you do add it and it's incorrect, it could be extremely frustrating. We are not meant to be looking at it. It could be very useful to have time stamps at each place so we could analyze at later time.	1.4	8%
1.3	Show real‐time refresh indicators and last‐updated timestamps per panel/table.	The screen is updated whenever anything changes. We do not feel a refresh indicator is needed. Discussed possibility of having a column for each dashboard that shows the date/time for the last time the status changed for each patient.	0	It would be nice to have this because everyone nowadays looking avidly at the screen. I've tried to upload a CT scan and nothing was uploaded and it doesn't tell you a time out. I initially scored this high.	1.3	0%
1.4	Provide filter pills that reflect active state (e.g., ‘Errors (5)’, ‘Awaiting Review (12)’).	Job status is shown. For errors that the user can influence (CT upload), hovering over the error shows more detail. Question: Do the users realize this, or do we need to improve clarity as not all items have the hover feature enabled.	2	One hospital right now has a whiteboard, and they manually write these down, but we found that this wasn't helpful. Another hospital says we need it because it's hard to see everything.	1.8	8%
1.5	Show background task progress with completion estimates.	We feel this is not needed, as the user is not expected to watch as tasks complete	0	Could be an indicator when something is taking longer than it should.	1.0	8%
1.6	Timestamps reassure users system is active and current.	See item 3 above	0	[no additional comments]	1.2	0%
1.7	Use color‐coded or symbolic cues to differentiate plans clearly. Color and symbolic cues ensure quick recognition and prevent mix‐ups.	Statuses are color‐coded, and symbols are used to indicate complete, in‐progress, waiting for the user, and so forth. Question: Do users think the colors and symbols we have are sufficient/appropriate?	1	Where do you draw the line—could be too many options.	0.6	0%

**TABLE 5:** Informative feedback: Suggestions and comments. High‐severity items are shown in red.

	ChatGPT suggestions	Developer comments	Developer group score	User comments	Average user group score	% scores ≥3
2.1	Detailed toasts (messages) with item counts and failure reasons ensure users know exactly what the system did after an action. Without this, uncertainty may cause duplicate actions or missed errors.	We currently only have detailed messages for things that may fail but the user can resolve (e.g., uneven CT slice‐spacing). Is this clear to the user? We may need to make it clearer where they can see this, for example, having an ‘i’ near the ‘X’ indicating that hovering over the symbol will give more information.	2	[no additional comments]	1.7	8%
2.2	Use inline, contextual checkmarks and timestamps next to each item updated.	See Table 4/item 3	0	[no additional comments]	1.2	0%
2.3	Provide audible cues and toast messages for long‐running tasks completion.	Not wanted for this application	0	[no additional comments]	0.2	0%
2.4	Offer download/view of upload logs for transparency.	We feel everything is visible on the dashboards. Question: Do the users agree?	0	If there was an audit, then we would need a way to do this. Being able to view history and see if there are mistakes and who used it is valuable. Perhaps having this functionality for the super‐user would be helpful.	2.1	38%

**TABLE 6:** Clear closure: Suggestions and responses.

	ChatGPT suggestions	Developer comments	Developer group score	User comments	Average user group score	% scores ≥3
3.1	Show a completion banner when the queue reaches zero, with counts by outcome.	We feel it is easy for the user to see on the dashboard when all jobs are complete. Queston: Do the users agree?	1	[no additional comments]	0.8	0%
3.2	Provide a ‘Triage complete’ summary card with timestamps and the responsible user.	We could add a column showing who submitted the job (physician or another person?). The user could then search by this. Question: Useful?	1	Irrelevant. It's not to say that the person who uploaded it will continue it.	0.5	0%
3.3	Offer a ‘Proceed’ CTA to the next logical workflow (e.g., notify stakeholders).	We do not feel this is useful but would like the users to clarify if they think the workflows are sufficiently obvious.	1	Tygerberg would like email notification connected. This may spam the doctor, and they won't read emails anymore. The users know what should happen.	1.6	15%
3.4	Provide explicit end‐of‐task confirmation banners.	Not needed. Already obvious.	0	[no additional comments]	0.5	8%

**TABLE 7:** Reversible actions: Suggestions and responses. High‐severity items are shown in red.

	ChatGPT suggestions	Developer comments	Developer group score	User comments	User group score	% scores ≥3
4.1	Provide a 5–10‐min undo window for Approve/Reject with versioned rollback. Time‐limited reverts support safe experimentation without lasting damage.	Possibly allow user to cancel after Service Request/CT accepted. Currently they have to wait for the entire job to be completed. It's not always easy to cancel stalled jobs—that could be useful	2	Right now, the way to undo it is to copy and delete. That time limit is not something I like.	0.8	8%
4.2	Add a ‘Reopen Request’ action that restores prior state and logs the change.	The ‘copy’ feature allows this	0	It may need an indicator if there are multiple plans. One option is to notify the user upon download—it could say what do you want to download and show you the like plans. Is there a way to show intent for each plan? Retire?	1.9	31%

**TABLE 8:** Users in control: Suggestions and responses.

	ChatGPT suggestions	Developer comments	Developer group score	User comments	User group score	% scores ≥3
5.1	Save draft, pause, and cancel affordances are needed so clinicians can manage interruptions. Healthcare environments are unpredictable, so workflows must allow flexibility.	Currently, partially complete Service Requests cannot be saved. We could add this feature. Question to users: Is it needed?	2	Right now, there are save functions. Would that allow multiple users to bring up the paused thing? You may pick up the wrong patient and make a mistake. It's better to do in one go. It may be better to force the user to get it done. It's risky.	0.8	8%
5.2	Expose ‘Save draft’, Pause’, and ‘Cancel’ everywhere; allow reordering and batching of permissible steps.	Currently, there is no cancel option. We could add this. See Table 7/item 1.	2	If a patient was more urgent, it may be useful, but this is cosmetic.	0.7	0%
5.3	User‐configurable Quick Actions empower clinicians to shape the dashboard to their common tasks, reducing time pressure and the chance of mistakes.	Possible solutions include filter by the patients per physician or other user, and going straight to the Service Request dashboard when physicians log in.	1	On aria you can customize the interface. If you are a doctor and you have logged in, you don't know what is ready. This is unnecessary when you spend so little time on the system. Could you start dashboard with cases in the last 30 days? Or it would be nice to display how many recent you would like displayed. It would be great to have user groups like if the GYN doc logged in and they see only GYN.	1.2	0%
5.4	Offer user settings to choose which comparison metrics are displayed.	Customizing the plan report? This is possible, but would it be useful? The user can (and should) do their own evaluation in their own treatment planning system	1	You could put this in as a customizable list—this could be useful.	1.9	8%
5.5	Enable layout customization (side‐by‐side vs. overlay view).	We do not want to allow this, as customization increases risk	0	[no additional comments]	0.9	8%
5.6	Persist for user‐selected preferences across sessions for consistency.	This seems reasonable. question: Do the users need this feature?	1	Most users are only doing one kind of plan at a time	1.1	0%

**TABLE 9:** Prevent errors: Suggestions and responses. High‐severity items are shown in red.

	ChatGPT suggestions	Developer comments	Developer group score	User comments	User group score	% scores ≥3
6.1	Block progression with clear, actionable preflight checks (orientation, slice spacing, reference points).	Preflight checks are built into all processes, with multiple questions and approvals. No action needed.	0	When reference point for breast is put at the wrong point, then there is no indication, only an error. One user: I don't like the block progress, but warning could be helpful.	1.5	38%
6.2	Add inline validation messages adjacent to offending fields with precise guidance—help users fix errors immediately instead of hunting for problems later.	Review of the interface indicates that the use of ‘i’ to show hover messages is inconsistent. May need to improve consistency.	2	Where would you want this where it is not already? It may be useful if you've created SR with no CT yet. So, seeing if it is matched or not would be helpful. There is a problem because some CTs group initial and first name differently and this causes conflicts in matching. We had to change the settings on the CT scanner (Toshiba) to show how to set the ordering of the names this makes association problem. Sometimes a special character has to be used to figure out surname versus initial. They had to repeat the anonymization to get it right.	1.5	15%
6.3	Implement safe defaults and constrained inputs (e.g., verified orientations, required fields) with preview.	All user‐entry is restricted by acceptable ranges. Question: Are there other changes we should make?	0	None	1.3	0%

#### 3.2.1 Visibility of system state

**Visibility of system state**. Definition (from Zhang^8^): Users should be informed about what is going on with the system through appropriate feedback and display of information.

- What is the current state of the system?
- What can be done at current state?
- Where can users go?
- What change is made after an action?

ChatGPT Comments: Global and per‐request status (e.g., whether uploads were validated, auto‐contours completed, or plan generation succeeded) is not persistently summarized. Counts by state (pending/approved/rejected) and last‐updated times are not visible at a glance, risking missed backlogs or acting on stale data. Without clear, persistent indicators and progress/breadcrumbs, users may misinterpret dashboard state and proceed prematurely.

#### 3.2.2 Informative feedback

**Informative feedback**. Definition (from Zhang^8^): Users should be given prompt and informative feedback about their actions.

- Information that can be directly perceived, interpreted, and evaluated.
- Levels of feedback (novice and expert).
- Concrete and specific, not abstract and general.
- Response time.
- 0.1 s for instantaneously reacting;
- 1.0 s for uninterrupted flow of thought;
- 10 s for the limit of attention.

ChatGPT comments: Key actions on the dashboard (e.g., launching a case, bulk operations) do not consistently produce specific, immediate confirmations with details and next‐step affordances, increasing ambiguity about outcomes.

#### 3.2.3 Clear closure

**Clear closure**. Definition (from Zhang^8^): Every task has a beginning and an end. Users should be clearly notified about the completion of a task.

- Clear beginning, middle, and end.
- Complete 7‐stages of actions.
- Clear feedback to indicate goals are achieved and current stacks of goals can be released. Examples of good closures include many dialogues.

ChatGPT comments: Completion of triage/processing is not clearly acknowledged with summaries or ‘all clear’ states; users may be unsure whether it's safe to move on. Workflow does not provide a clear ‘upload complete’ summary or confirmation banner.

Resolving these are necessary because:

- Clear closure prevents over‐checking or premature transitions to other tasks.
- Summaries and banners document completion for accountability and audits.
- Explicit ‘Proceed’ steps reduce hesitation and keep workflows efficient.

#### 3.2.4 Reversible actions

**Reversible action**s (undo). Definition (from Zhang^8^): Users should be allowed to recover from errors. Reversible actions also encourage exploratory learning.

- At different levels: a single action, a subtask, or a complete task.
- Multiple steps.
- Encourage exploratory learning.
- Prevent serious errors

ChatGPT comments: Critical actions appear irreversible from the dashboard context; no short‐window undo, no ‘Reopen’ option, and limited confirmation before bulk changes, raising recovery cost for slips.

#### 3.2.5 Users in control

**Users in control**. Definition (from Zhang^8^): Do not give users that impression that they are controlled by the systems.

- Users are initiators of actors, not responders to actions.
- Avoid surprising actions, unexpected outcomes, tedious sequences of actions, and so forth.

ChatGPT comments: The dashboard flow feels system‐gated with few options to pause, cancel, or safely stage changes—users may feel the system is in control rather than them, especially during long‐running updates. Users have limited control, for example, cannot prioritize or reorder multiple uploads, and lack ability to pause/resume. Limited user control over how plans are displayed and compared, lack of customizable comparison metrics or view persistence.

#### 3.2.6 Prevent errors

**Prevent errors**. Definition (from Zhang^8^): It is always better to design interfaces that prevent errors from happening in the first place.

- Interfaces that make errors impossible.
- Avoid modes (e.g., vi, text wrap). Or use informative feedback, for example, different sounds.
- Execution error versus evaluation error.
- Various types of slips and mistakes.

ChatGPT comments: There is insufficient visible validation/guardrails for common slips (e.g., wrong orientation, missing reference points) before proceeding. Strong affordances to prevent errors up front are essential in clinical contexts.

## 4 DISCUSSION

This study evaluated whether ChatGPT could meaningfully augment a heuristic review of the Radiation Planning Assistant (RPA). Using Zhang's 14 heuristics, ChatGPT generated 26 interface suggestions spanning visibility, feedback, closure, reversibility, user control, and error prevention. Out of these, developers scored nine suggestions actionable, all minor in severity, while user scores showed wide variability and poor agreement with developers. A minority of users highlighted three potential priorities: better upload/audit logs, a reversible “reopen request” action, and clearer preflight checks to prevent errors. No critical failures were identified, consistent with RPA's prior usability work and shared design across dashboards. ChatGPT's ratings were consistent across dashboards, probably reflecting the shared design lineage of RPA modules and the prior usability testing performed before clinical deployment. Overall, ChatGPT's suggestions promoted constructive discussion and helped surface incremental refinements, supporting its role as a low‐cost complement to traditional usability engineering in safety‐relevant medical software.

The suggestions which users were more likely to identify as necessary (although still a minority of users):

- Informative feedback: Offer download/view of upload logs for transparency. Further investigation after the meeting found that the users can request logs—inspection of these logs showed that they are adequate.
- Reversible actions: Add a ‘Reopen Request’ action that restores prior state and logs the change. This complements the existing “copy and delete” workflow and may reduce recovery cost for slips without encouraging risky exploration.
- Prevent errors: Block progression with clear, actionable preflight checks (orientation, slice spacing, reference points). The RPA has many preflight checks, but we need to investigate adding more.

Importantly, discussions at the users meeting that were seeded by the ChatGPT suggestions, followed by review by the RPA Team, led to 7 items being added to the Change Request Log, part of the RPA team's quality management system. These will be regularly reviewed for potential system improvements.

The divergence between developer and user ratings is consistent with the subjectivity inherent to heuristic evaluation, particularly in safety relevant domains where clinical context, mental models, and institutional practices shape perceived priorities. Developers—who are more familiar with design intent, existing guardrails, and backend constraints—tended to classify issues as cosmetic or low priority, whereas several users emphasized different usability issues. Importantly, average scores were modest (≈1.1–1.3 with SD ≈0.5–0.6), indicating broad agreement that the proposed changes were not fundamentally essential usability issues.

Our observations echo prior expert and user evaluations of radiotherapy treatment planning and delivery systems, which frequently surface issues in feedback clarity, workflow visibility, and error prevention but seldom identify catastrophic failures in mature products.^9^, ^25^ Chan et al. and Jiang et al. reported that many usability problems in radiation oncology software cluster around information presentation (e.g., status visibility, message clarity), navigation, and error prevention—categories that also dominated ChatGPT's suggestions for RPA. Relative to traditional expert panels, the LLM added value by rapidly generating structured, context aware prompts that catalyzed discussion among developers and end users. Within the broader apparatus of medical device usability engineering recommended by IEC 62366, our results suggest that LLM assisted heuristic review can serve as a low cost complement rather than a replacement for established methods.

This work focused on ChatGPT, a readily available LLM. There are, of course, many other LLMs (Gemini, Claude, Llama‐3, etc.), each with different strengths and limitations—for example different reasoning quality, hallucination rates, and overall performance. Although interesting, comparison of the different models was outside the scope of this work, although it could be a topic for future research. That said, the extremely rapid development of LLMs mean that any claims about different LLMs will soon be outdated. Our overall conclusion that LLMs can support heuristic analysis, however, should not change.

One potential limitation of this work is that ChatGPT was unable to interact with the actual system in real time, and had to perform analysis based on documentation and video frames. ChatGPT could not assess each training video in its entirety, but was restricted to 10 evenly spaced frames (as well as the user documentation), which could lead to interpretation errors. This number was chosen iteratively, to cover significant aspects of the videos without overwhelming ChatGPT. Manual review of the selected frames indicated that this was reasonably successful in covering the main tasks covered in each video. Other limitations include that ChatGPT's evaluation was based on documentation, screenshots, and training videos rather than unrestricted, interactive use; some workflow‐contingent issues (e.g., edge‐case navigation, time pressure, interruptions) may therefore have been missed. Another limitation of this approach is that ChatGPT's feedback may reflect patterns in its training data rather than a true application of heuristic principles. Other limitations are related to the raters, such as the somewhat limited sample size (13), potential bias (all from the same country etc.), that many of the Users had only limited experience of the RPA, and that there was only one radiation oncologist present at the meeting.

Beyond the use of LLMs to support heuristic evaluation, LLMs can support other aspects of clinical software assessment. For example, generating error scenarios, highlighting workflow inconsistencies, and evaluating clarity of interface terminology or documentation. This work has demonstrated some lessons that are likely to be relevant to these other applications, such as the need for oversight, and the use of the LLM to seed discussions between users.

## 5 CONCLUSIONS

ChatGPT's heuristic evaluations provided meaningful input for identifying potential usability refinements to the RPA. While most issues identified were of low severity, the exercise demonstrated the utility of large language models as a complementary resource in usability assessment, particularly for generating discussion and highlighting areas for reflection among developers and users. As such, LLM‐assisted heuristic evaluation is complementary to standard usability testing, not a substitute. This study provides a process for using LLMs in heuristic usability analysis, especially for encouraging user interaction and feedback.

## AUTHOR CONTRIBUTIONS

All authors contributed to the implementation of the research, data collection, the analysis of the results, and writing the manuscript.

## CONFLICT OF INTEREST STATEMENT

The Radiation Planning Assistant project has been funded by the NI/NCI, Varian Medical Systems, Cancer Prevention Research Institute of Texas, Wellcome Trust and the University of Texas MD Anderson Cancer Center.

## Data Availability

All data are presented in this paper.

## USABILITY SURVEY FROM THE USERS MEETING

### 1

**Mini‐Project Workshop: Using AI to Guide Usability Testing of Medical Software**

**Name**:

**Department and Institution**:

I have used the RPA: yes / no

**Instructions**

Please score each ChatGPT usability suggestion using the score below.

If you have additional thoughts or suggestions about usability, please (in each section, or at the end).

**Scoring**

0 = Not a usability problem at all

1 = Cosmetic problem only. Need not be fixed unless extra time is available

2 = Minor usability problem. Fixing this should be given low priority

3 = Major usability problem. Important to fix. Should be given high priority

4 = Usability catastrophe. Imperative to fix before product release

**Visibility of system state**.

Users should be informed about what is going on with the system through appropriate feedback and display of information.

- What is the current state of the system?
- What can be done at current state?
- Where can users go?
- What change is made after an action?

**Table:**

#	Description	Usability score
1	Add a persistent global status area (system health, queue length)	
2	Display real‐time indicators (spinners, timestamps, percentage complete)	
3	Show real‐time refresh indicators and last‐updated timestamps per panel/table	
4	Provide filter pills that reflect active state (e.g., ‘Errors (5)’, ‘Awaiting Review (12)’)	
5	Show background task progress with completion estimates	
6	Timestamps reassure users system is active and current	
7	Use color‐coded or symbolic cues to differentiate plans clearly. Color and symbolic cues ensure quick recognition and prevent mix‐ups	

**Informative feedback**.

Users should be given prompt and informative feedback about their actions.

- Information that can be directly perceived, interpreted, and evaluated.
- Levels of feedback (novice and expert).
- Concrete and specific, not abstract and general.
- Response time.

• 0.1 s for instantaneously reacting;
• 1.0 s for uninterrupted flow of thought;
• 10 s for the limit of attention.

**Table:**

#	Description	Usability score
1	Detailed toasts (messages) with item counts and failure reasons ensure users know exactly what the system did after an action. Without this, uncertainty may cause duplicate actions or missed errors	
2	Use inline, contextual checkmarks and timestamps next to each item updated	
3	Provide audible cues and toast messages for long‐running tasks completion	
4	Offer download/view of upload logs for transparency	

**Clear closure**.

Every task has a beginning and an end. Users should be clearly notified about the completion of a task.

- Clear beginning, middle, and end.
- Complete 7‐stages of actions.
- Clear feedback to indicate goals are achieved and current stacks of goals can be released. Examples of good closures include many dialogues.

**Table:**

#	Description	Usability score
1	Show a completion banner when the queue reaches zero, with counts by outcome	
2	Provide a ‘Triage complete’ summary card with timestamps and the responsible user	
3	Offer a ‘Proceed’ CTA to the next logical workflow (e.g., notify stakeholders)	
4	Provide explicit end‐of‐task confirmation banners	

**Reversible action**s (undo).

Users should be allowed to recover from errors. Reversible actions also encourage exploratory learning.

- At different levels: a single action, a subtask, or a complete task.
- Multiple steps.
- Encourage exploratory learning.
- Prevent serious errors

**Table:**

#	Description	Usability score
1	Provide a 5–10 min undo window for Approve/Reject with versioned rollback. Time‐limited reverts support safe experimentation without lasting damage	
2	Add a ‘Reopen Request’ action that restores prior state and logs the change	

**Users in control**.

Do not give users that impression that they are controlled by the systems.

- Users are initiators of actors, not responders to actions.
- Avoid surprising actions, unexpected outcomes, tedious sequences of actions, and so forth.

**Table:**

#	Description	Usability score
1	Save draft, pause, and cancel affordances are needed so clinicians can manage interruptions. Healthcare environments are unpredictable, so workflows must allow flexibility	
2	Expose ‘Save draft’, ‘Pause’, and ‘Cancel’ everywhere; allow reordering and batching of permissible steps	
3	User‐configurable Quick Actions empower clinicians to shape the dashboard to their common tasks, reducing time pressure and the chance of mistakes	
4	Offer user settings to choose which comparison metrics are displayed	
5	Enable layout customization (side‐by‐side vs. overlay view)	
6	Persist user‐selected preferences across sessions for consistency	

**Prevent errors**.

It is always better to design interfaces that prevent errors from happening in the first place.

- Interfaces that make errors impossible.
- Avoid modes (e.g., vi, text wrap). Or use informative feedback, for example, different sounds.
- Execution error versus evaluation error.
- Various types of slips and mistakes.

**Table:**

#	Description	Usability score
1	Block progression with clear, actionable preflight checks (orientation, slice spacing, reference points)	
2	Add inline validation messages adjacent to offending fields with precise guidance—help users fix errors immediately instead of hunting for problems later	
3	Implement safe defaults and constrained inputs (e.g., verified orientations, required fields) with preview	

**Suggestions for improvement of the user interface, including workflows**

________________________________________________________________________________________________________________________________________________
